# Culture-independent detection of *Mycobacterium tuberculosis* complex DNA using targeted next generation sequencing in African buffalo (*Syncerus caffer*) oronasal swabs in South Africa

**DOI:** 10.3389/fvets.2025.1523628

**Published:** 2025-02-07

**Authors:** Sinegugu Kholeka Mhlophe, Charlene Clarke, Giovanni Ghielmetti, Megan Matthews, Tanya Jane Kerr, Michele Ann Miller, Wynand Johan Goosen

**Affiliations:** ^1^Division of Molecular Biology and Human Genetics, Faculty of Medicine and Health Sciences, SAMRC Centre for Tuberculosis Research, Stellenbosch University, Stellenbosch, South Africa; ^2^Section of Veterinary Bacteriology, Vetsuisse Faculty, Institute for Food Safety and Hygiene, University of Zurich, Zurich, Switzerland; ^3^Department of Microbiology and Biochemistry, Faculty of Natural and Agricultural Sciences, University of the Free State, Bloemfontein, South Africa

**Keywords:** African buffaloes, culture-independent detection, *Mycobacterium tuberculosis* complex, oronasal swabs, Oxford Nanopore Technologies, targeted next generation sequencing

## Abstract

African buffaloes (*Syncerus caffer*) are wildlife maintenance hosts of *Mycobacterium bovis* (*M. bovis*), the causative agent of animal tuberculosis (aTB) in multiple ecosystems across South Africa. In addition to their role as keystone species, these animals are vital to South Africa’s economy as a highly valuable species. Controlling aTB in South Africa relies on mycobacterial culture as the gold standard for *M. bovis* confirmation, with the single intradermal comparative cervical test (SICCT) and Bovigam™ assays as validated cell-mediated immunological assays for detection. However, these methods are not without their shortfalls, with a suboptimal ability to discern true positive results amidst certain non-tuberculous mycobacteria (NTM) interference. This study employed a culture-independent approach using oronasal swabs collected from African buffaloes (*n* = 19), originating from three herds with no recorded history of *M. bovis* infection, to elucidate the possible cause of observed discordant immunological aTB test results. The DNA was extracted directly from the oronasal swabs, amplified using *Mycobacterium* genus-specific PCRs, then amplicons were pooled and sequenced using Oxford Nanopore Technologies (ONT) long-read platform. *Mycobacterium tuberculosis* complex DNA, along with various NTM species, were identified in 8/19 samples. The methods described support a more robust interrogation of the buffalo oronasal mycobacteriome. These findings highlight the value of accurately distinguishing between mycobacterial species in complex samples, especially in high-value animals, to facilitate accurate interpretation of immunological test results and management of aTB.

## Introduction

African buffaloes (*Syncerus caffer*) are well-known wildlife maintenance hosts for *Mycobacterium bovis* (*M. bovis*) in various national parks and game reserves across southern Africa (1, 2). In South Africa (SA), these include the Hluhluwe-iMfolozi Park (HiP) and Kruger National Park (KNP), where *M. bovis* is endemic (3). African buffaloes provide vital ecological functions as keystone species in these ecosystems (4–6). Additionally, they are important for ecotourism, hunting, and game sales, which are significant economic contributors to the wildlife industry in SA (4, 7). However, the presence of *M. bovis* in buffaloes can result in the quarantine of farms or parks, with restrictions on movement, trade, and potential culling of genetically valuable individuals (4). Early detection and control of *M. bovis* infections in African buffaloes can prevent its introduction through translocation, reduce inter- and intra-species transmission, and reduce economic loss associated with animal tuberculosis (aTB).

Although antemortem methods for detecting *M. bovis* infection have improved, mycobacterial culture and speciation, usually from postmortem tissue samples, continues to be the gold standard for diagnosing infection (8). Positive cultures require further downstream genetic speciation, commonly using PCR-based methods targeting areas such as region-of-difference (RD) or insertion elements for *Mycobacterium tuberculosis* complex (MTBC) identification (8, 9). However, in the absence of tissue samples, antemortem samples, such as oronasal, respiratory, or fecal samples, are often used for mycobacterial culture, especially for diagnosing high-value individuals.

Respiratory samples are typically used for antemortem direct detection of MTBC (8, 10, 11). Oronasal swabs are easier to collect, less invasive and inexpensive when compared to obtaining respiratory samples using bronchioalveolar lavage (12). However, samples from the oronasal cavity often contain a variety of microbial contaminants, including environmental non-tuberculous mycobacteria (NTM), and results may represent shedding from an infected animal as well as oronasal colonization (9, 13). Although culture can detect viable *M. bovis* within 6–8 weeks, rapidly growing mycobacteria often outcompete slow-growing organisms *in vitro* for nutrients and oxygen, subsequently impeding detection of MTBC in paucibacillary samples, leading to false-negative culture results (14, 15). Consequently, the conventional culture approach may not accurately identify all mycobacteria present in complex samples, especially in samples with low MTBC abundance and heterogeneous mycobacterial populations. Thus, accurate characterization of MTBC and NTMs in respiratory samples is crucial for a correct diagnosis of aTB (15–17). This will require improved detection and characterization techniques to identify MTBC in paucibacillary complex samples.

Conventional mycobacterial culture, followed by PCR and amplicon sequencing, have been reported for speciating mycobacteria, although there are still limitations using this approach (16–18). Recently, a culture-independent approach, using Oxford Nanopore Technologies (ONT) targeted next generation sequencing (tNGS) of DNA extracted from bronchoalveolar lavage fluid (BALF), has shown superior sensitivity for MTBC detection compared to culture (10). This method has also been used with *M. bovis* infected buffalo tissue, with ONT tNGS results consistent with results from culture (17). These promising results suggest that ONT tNGS could be used in time-sensitive cases or for a more detailed characterization of mycobacterial species and strains in clinical samples. Therefore, this study aimed to characterize the oronasal mycobacteriome of African buffalo culture-independently using ONT tNGS, comparing results to previously determined mycobacterial culture outcomes, to assess its potential as a complementary technique to identify MTBC, and NTMs that may lead to discordant immunological test results.

## Materials and methods

### Ethical clearance

Ethical approval for African buffalo sampling was obtained from Stellenbosch University’s (SU) Animal Care and Use Committee (ACU-2019-9081 and ACU-2019-9086). Permission for animal research was granted by the South African Department of Agriculture, Land Reform, and Rural Development (DALRRD) under Section 20 of the Animal Diseases Act (12/11/1/7/2).

### Study cohort and selection criteria for inclusion

**Figure 1:**
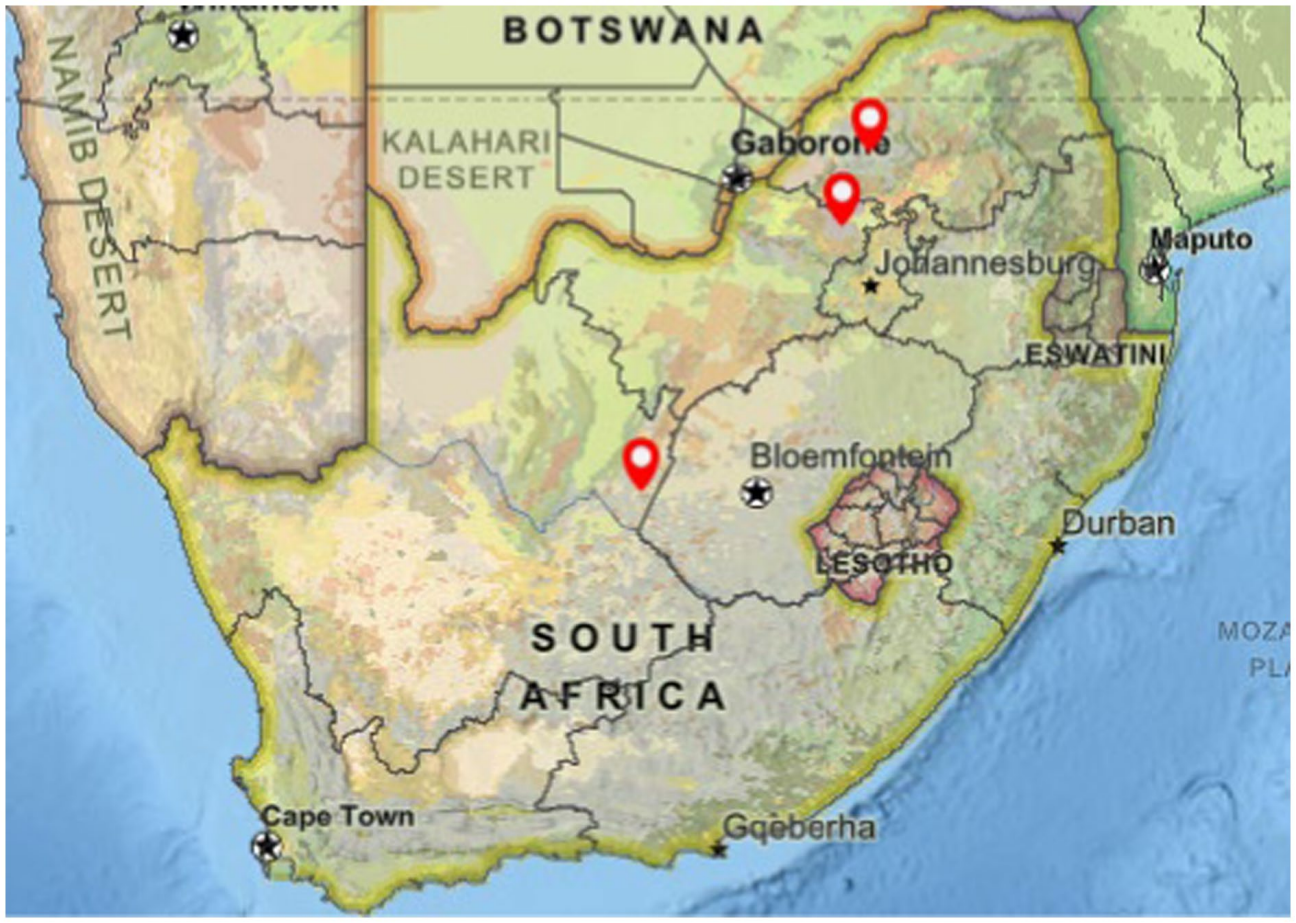
Map of South Africa with balloons showing the locations where African buffalo (*Syncerus caffer*) herds were sampled for this study. Oronasal swabs were collected from immobilized animals, located in the Northern Cape, North-West, and Limpopo provinces, in wildlife reserves where *Mycobacterium bovis* (*M. bovis*) infection had not been previously detected. Image created with National Geographic MapMaker (arcgis.com).

Two oronasal swabs were collected from each of 120 buffalo, as part of a previous study (19), to characterize the diversity of NTMs present in four African buffalo herds in South Africa with no previous herd history of *M. bovis* infection. In addition, blood was collected for antigen-specific cytokine release assays. Despite the herds’ TB-free status, several buffalo from three herds (Figure 1) showed immunological responses to mycobacterial antigens, based on either the Single Intradermal Comparative Cervical Tuberculin (SICCT) test, QuantiFERON®-TB Gold Plus (QFT) bovine interferon gamma (IFN-*γ*), or QFT bovine interferon gamma-induced protein 10 (1P-10) release assays. A subset of these individuals (*n* = 19) was selected to investigate the presence of MTBC and NTM associated with the immune sensitization responses (19). Briefly, mycobacterial culture as well as direct DNA extraction of oronasal swabs were performed, followed by heat shock protein of 65 kDa (*hsp65*) and partial RNA polymerase beta subunit (*rpoB*) PCR amplicon Sanger sequencing (19). Sequences with ≥90% sequence identity match were used to identify NTM species (in the absence of MTBC) in 19 oronasal swab samples (19). The oronasal swabs were further evaluated using culture-independent ONT tNGS to identify and determine the relative abundance of mycobacterial species for comparison with the results of the previous study (19). Postmortem samples were not available for the study cohort. For the current study, the previous swab culture Sanger sequences for *hsp65* and *rpoB* were re-analyzed to apply stricter coverage and percentage identity thresholds, as described below. This was performed to ensure that the results obtained were comparable to those obtained with ONT tNGS.

### DNA extraction from oronasal swabs, *Mycobacterium* spp. PCR amplification, and sanger sequencing

Oronasal swab samples from African buffalo (*n* = 19) were subjected to DNA extraction using the DNeasy Blood & Tissue Kit (QIAGEN, Hilden, Germany), as previously described (20). Initial screening for *Mycobacterium* spp. DNA was accomplished using PCR amplification of the *hsp65* and *rpoB* genes (Table 1), as previously described (19). Briefly, each PCR reaction had a total volume of 25 μL and contained 12.5 μL of Q5® Hot Start High-Fidelity 2X Master Mix (New England Biolabs, Ipswich, MA, USA), 1 μL of forward primer and 1 μL of reverse primer at 10 μM, 2 μL of extracted DNA, and 8.5 μL of nuclease-free water (Thermo Fisher Scientific™, Waltham, MA, USA). Nuclease-free water and *M. bovis* DNA were used as the negative and positive controls, respectively. The thermal cycling program included an initial denaturation step at 98°C for 15 min. This was followed by 35 cycles, each consisting of denaturation at 98°C for 30s, annealing at 62.5°C (for *hsp65*) or 64°C (for *rpoB*) for 30s, and extension at 72°C for 1 min. The final reaction step was performed at 72°C for 5 min. The presence of amplicons was confirmed by visualization on a 1% agarose gel, and Sanger sequencing was performed at Stellenbosch University’s Central Analytical Facility (CAF). Sanger sequences were assembled into contiguous sequences (contigs) using ApE-A Plasmid Editor version 3.1.5 (21). The resulting contigs were aligned with *Mycobacterium* spp. sequences, using the NCBI Nucleotide Basic Local Alignment Search Tool (BLASTn) (47). Genus was assigned to samples that had Sanger sequences with ≥90% similarity to the reference sequence, with a minimum sequence coverage of 90%. The most likely mycobacterial species identity for each sequence was selected based on ≥99% similarity to the reference sequence, with a minimum coverage of 90%. The same criteria were applied with other genera identified.

**Table 1:** Oligonucleotide primer sequences (Integrated DNA Technologies, Coralville, IA, USA), resulting amplicon size for *Mycobacterium* spp. genes, and annealing temperatures for PCR amplification of DNA extracted directly from African buffalo (*Syncerus caffer*) oronasal swabs.

Gene Target	Reference	Forward/Reverse	Primer sequence 5′-3’	Product size (bp)	T_a_ (°C)
*hsp65*	(43)	Forward	ACCAACGATGGTGTGTCCAT	441	62.5
		Reverse	CTTGTCGAACCGCATACCCT		
MAC*hsp65*	(22)	Forward	AATTGCGTACGACGAAGAGG	1,621	55
		Reverse	ACGGACTCAGAAGTCCATGC		
*rpoB*	(44)	Forward	GGCAAGGTCACCCCGAAGGG	739	64
		Reverse	AGCGGCTGCTGGGTGATCATC		
*gyrB1*	(45)	Forward	CGGCTCGAAGTCGAGATCAAG	144	55
		Reverse	TTCGAAAACAGCGGGGTCG		
*gyrB2*	(46)	Forward	CAAATCGTTTGTGCAGAAGGTCTG	107	55
		Reverse	CTTGCGCCGAGGACACAG		
*gyrA*	(46)	Forward	AGGCAATCCTGGACATGCAG	107	55
		Reverse	GATGTCTTCCAGATCGGCGATC		

### Oxford Nanopore Technologies (ONT) library preparation and targeted amplicon sequencing (tNGS)

Additional mycobacterial gene targets (Table 1) were selected, and swab DNA underwent ONT tNGS. *Mycobacterium avium* complex (MAC) *hsp65* (MAC*hsp65*) was used to detect the presence of MAC and other NTM (22). The target DNA gyrase subunits A and B (*gyrA, gyrB1,* and *gyrB2*) were amplified to confirm the presence of *M. bovis* and visualized on a 1% agarose gel, as described by Ghielmetti et al. (17). The DNA concentration of PCR amplicons for all amplified targets in each sample was quantified with the Qubit 1x dsDNA High Sensitivity (HS) Assay Kit (Thermo Fisher Scientific™). Equal amounts of 300 femtomoles of each target were pooled and prepared as a single barcoded library, using the ONT Native Barcoding Kit 96 v14 (ONT, Oxford Science Park, Oxford, UK), according to the manufacturer’s instructions. The pooled library was loaded onto primed flow cells, either R10.4.1 or Flongle (ONT), each with >1,400 and > 60 active pores, respectively. Each barcode represented a sample from one buffalo, which allowed simultaneous multigenetic sequencing using the ONT MinION Mk1C device (ONT).

For the targeted amplicon sequencing datasets, base-calling, demultiplexing, and trimming of the barcodes were performed in real time using Guppy [v6.4.6] (260 bps, high accuracy) (23). Data acquisition and base-calling were stopped after 16 h for the *hsp65, rpoB*, and MAC*hsp65* runs, using an R10.4.1 flow cell (FLO-MIN114, ONT), and after 17 h for the *gyrA* and *gyrB* runs, using a Flongle flow cell (FLO-FLG114, ONT) (Supplementary material S1). Quality control, filtering, and summary reports for Nanopore reads were generated using Nanoq v0.10.0 (24), and reads with a Q score of <12 were discarded. Following quality control checks, reference-free read sorting, based on similarity and length, was performed using Amplicon sorter (v2023-06-19) (25). A total of 200,000 randomly chosen reads with minimum and maximum lengths of 300 and 2,000 bp, respectively, were selected for each barcode generated using three target amplicons (*hsp65*, *rpoB*, and MAC*hsp65*). Finally, ABRicate and custom databases were used to screen consensus sequences and summarize the report files, as previously described (20).

Reads of *gyrA* and *gyrB* amplicons were selected based on a minimum length of 50 bp to a maximum of 200 bp. Consensus sequences were then generated for each identified target gene and bacterial genus/species. Relative abundance was determined by analyzing a representative pool of reads. Sequence comparison with a custom database, generated with *gyrA* and *gyrB* sequences for all MTBC members, was performed as described above. For all bioinformatics tools, default settings were used unless stated otherwise.

The ONT sequencing results were interpreted based on the same criteria used for Sanger sequences: only sequences with ≥90% coverage were considered for further analysis. Sequences with a percentage identity match <90% were considered unclassified. Sequences with a percentage identity match between 90 and 99% were reported at the genus level. Sequences with a percentage identity match of ≥99% were reported as the species listed in the database. The resulting high-quality sequenced reads were reported as described above. The relative abundance of reads for each identified genus/species was graphically summarized, where the number of reads was normalized by converting the values into percentages. Samples with MTBC DNA were further investigated to detect *M. bovis* DNA using RD4 PCR, as previously described (26).

## Results

### *Mycobacterium* spp. identification by sanger sequencing *hsp65* and *rpoB* PCR amplicons from mycobacterial culture and directly from oronasal swab DNA

Sanger sequencing of *hsp65* and *rpoB* PCR amplicons generated from DNA extracted directly from oronasal swabs or swab cultures (19) identified 13 and 12 out of 19 samples, respectively, as positive for *Mycobacterium* spp. at the genus level (90–99% identity match). When using a ≥ 99% identity match to assign species directly from the oronasal swabs or swab cultures, 5 and 2 out of 19 samples, respectively, could be speciated, with all species identified as *M. avium* (Supplementary materials S3). Bacteria other than *Mycobacterium* spp. with ≥99% identity match from both culture and swabs included *Cellulomonas* spp. (*n* = 2), *Rhodococcus* spp. (*n* = 1), and *Cellulosimicrobium* spp. (*n* = 1). No MTBC DNA was identified in any of the samples by PCR amplicon Sanger sequencing of the swab cultures, nor directly from the oronasal swabs.

### *Mycobacterium* spp. identification in oronasal swab DNA based on ONT tNGS of pooled PCR amplicons

Sequencing reads with Q scores >12 ranging from 14,310 to 262,898 per sample [mean (*M*) = 110,900; standard deviation (SD) = 79,866] were generated (Supplementary materials S1, S2). The number of bases ranged from 4,376,240 to 210,600,527 [*M* = 66,579,718; SD = 57,957,100]. The median read length of the longest contigs (N50 read length) spanned 268–881 reads. The Phred score (indicative of the average read quality) was between 13.1 and 14.8 per sample. The distribution of the number of reads per PCR target is presented in Table 2. Twelve oronasal swab samples contained *Mycobacterium* spp. (identity match ≥99%), based on *hsp65* and *rpoB* ONT tNGS (Supplementary materials S3).

**Table 2:** Distribution of read counts per PCR amplification target used to identify mycobacteria in DNA extracted from African buffalo (*Syncerus caffer*) oronasal swabs (*n* = 19), based on sequence coverage and identity match at ≥90%.

Target	Read Count	No. of Samples identified with *Mycobacterium* spp.
*hsp65*	445,689	13
*rpoB*	97,536	13
MAC*hsp65*	48,783	11
*gyrB*	71,592	5

Base-calling and read generation occurred for 16-17 h using Oxford Nanopore Technologies flow cells and the Mk1C device. Reads generated were between 66 and 2000 bp long and all reported samples passed quality control (Q score > 12).

**Figure 2:**
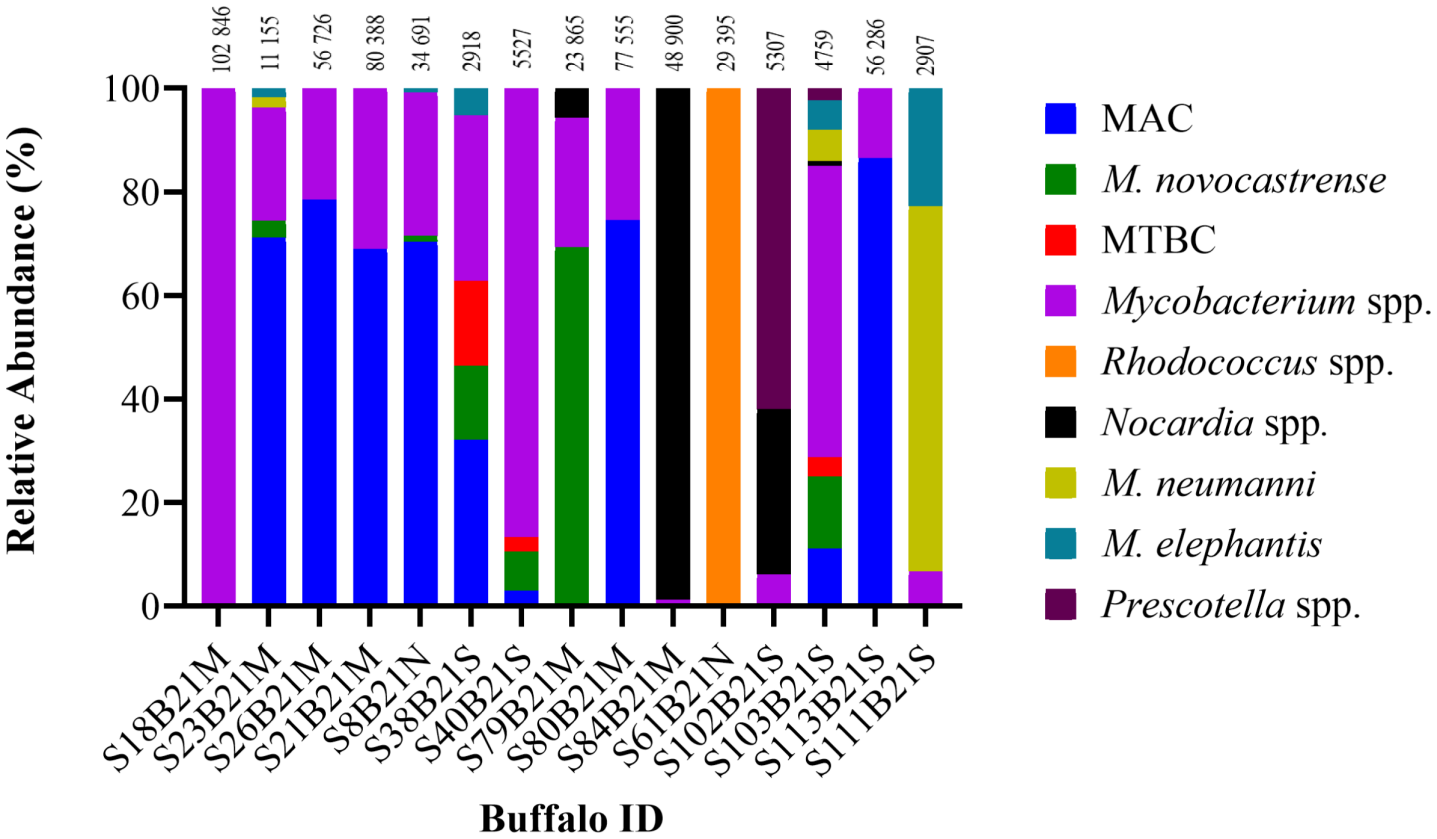
Distribution of the relative abundance of *hsp65* and *rpoB* amplicon reads sequenced from 15 out of 19 DNA samples extracted from African buffalo (*Syncerus caffer*) oronasal swabs using the MinION Mk1C device (ONT). Four DNA samples failed to generate amplicons from *hsp65* and *rpoB* PCRs and were excluded from the figure. Relative read abundance was based on sequences with a ≥ 90% identity match to reference sequences, with the number above each bar representing the total number of reads assigned to each sample at this threshold. The MTBC DNA in sample S40B21S was identified as *M. bovis* by RD4 PCR; the remaining two MTBC samples (S38B21S, S103B21S) could not be speciated. Reference-free sorting and assembly of consensus sequences were performed using a maximum of 200,000 randomly selected reads (> Q12) for each sample. Reads that could not be assigned were grouped as unclassified and excluded. MAC, Mycobacterium avium complex; MTBC, Mycobacterium tuberculosis complex.

**Figure 3:**
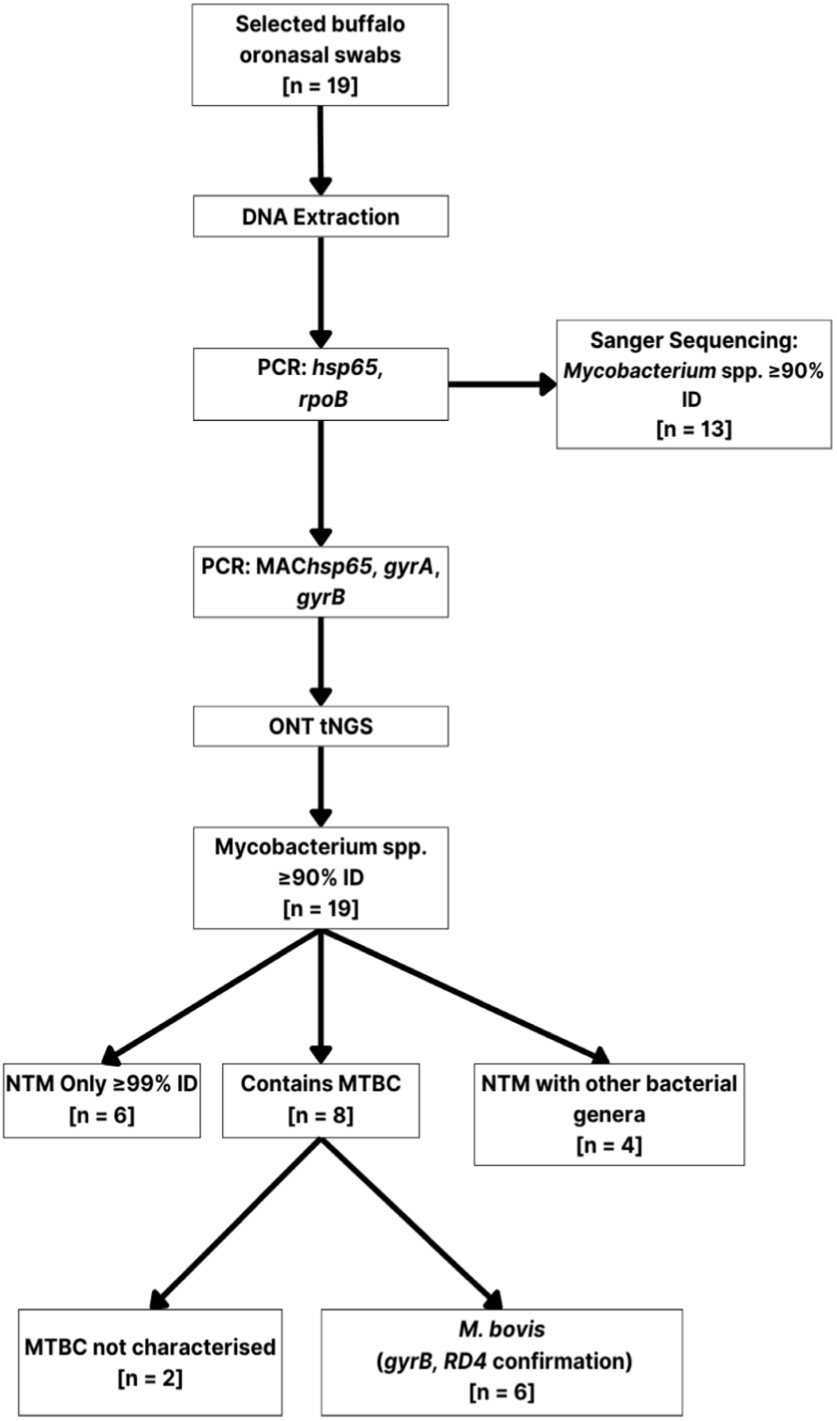
Branched diagram summarizing the culture-independent methods used to characterize mycobacteria present in African buffalo (*Syncerus caffer*) oronasal swabs (*n* = 19). Extracted DNA was subjected to multi-target PCR (*hsp65, rpoB,* MAC*hsp65, gyrA, gyrB,* RD4), Sanger sequencing, and ONT sequencing for mycobacterial speciation. Numbers (*n*) in boxes represent the number of samples assigned to that category. *Mycobacterium* spp. DNA was detected in samples with PCR amplicon Sanger sequences having ≥90% identity match (≥ 90% ID) with reference sequences. ONS, oronasal swab; PCR, polymerase chain reaction; NTM, non-tuberculous mycobacteria; ONT, Oxford Nanopore Technologies; MTBC, Mycobacterium tuberculosis complex.

The ONT tNGS results, using *hsp65*, *rpoB,* MAChsp*65,* and *gyrB* amplicon sequences with ≥99% identity match, showed that 17/19 samples contained *Mycobacterium* spp. DNA; 13 were identified using *hsp65* and *rpoB*, 11 with MAC*hsp65*, and 5 with *gyrB* (Table 2). Figure 2 shows the relative abundance of *hsp65* and *rpoB* amplicon reads sequenced from 15 out of 19 DNA samples extracted from African buffalo (*Syncerus caffer*) oronasal swabs, using the MinION Mk1C device (ONT). Four DNA samples failed to generate amplicons from *hsp65* and *rpoB* PCRs and were excluded from this figure. Based on the 15 *hsp65* and r*poB* amplicon sequences, 3 samples were identified as containing MTBC DNA (Figure 2). An additional five samples were confirmed to contain *M. bovis* DNA, based on *gyrB* sequencing, bringing the total to eight samples with MTBC DNA (Figure 3; Supplementary material S3). Speciation by RD4 PCR (based on 268 bp band on gel electrophoresis) confirmed an additional sample contained *M. bovis* DNA (identity assigned as MTBC by tNGS) (Figure 3; Table 3). Interestingly, DNA from 4 of the 5 *gyrB* positive samples could not be amplified using either *hsp65*, *rpoB,* or MAC*hsp65.* Therefore, based on multiple amplicon sequences, a total of 8 out of 19 samples that had DNA extracted directly from oronasal swabs contained MTBC DNA, with 6 speciated to *M. bovis* (Figure 3).

**Table 3:** Oxford Nanopore Technologies (ONT) targeted (*hsp65, gyrB*) next generation sequencing read count distribution (using the Mk1C device) and RD4 PCR results.

Buffalo ID	MTBC member	Target	No. of reads	RD4 PCR result
S40B21S	*M. bovis*	*hsp65*	158	268 bp
S47B21S	*M. bovis*	*gyrB*	7,640	268 bp
S55B21S	*M. bovis*	*gyrB*	11,094	268 bp
S39B21S	*M. bovis*	*gyrB*	16,845	268 bp
S111B21S	*M. bovis*	*gyrB*	22,951	268 bp
S115B21S	*M. bovis*	*gyrB*	13,062	268 bp

bp, base pairs.

Six out of eight African buffalo (*Syncerus caffer*) oronasal swab samples with *Mycobacterium tuberculosis* complex (MTBC) DNA detected using *hsp65* or *gyrB* amplicon sequencing, were confirmed to contain *Mycobacterium bovis* (*M. bovis*) DNA based on RD4 PCR results (268 bp band on gel electrophoresis).

In addition to MTBC DNA, NTMs were identified in 13 samples using *hsp65*, *rpoB,* and MAC*hsp65* tNGS (≥ 99% identity match), with the results from *hsp65* and *rpoB* shown in Figure 2. Using these three targets, oronasal swab DNA had matches with *M. avium* complex members (MAC) (10/13), *M. novocastrense* (5/13), *M. neumanni* (4/13), and *M. elephantis* (6/13), with variable abundance. In addition to NTMs (with or without MTBC), *Rhodococcus* spp. (*n* = 1) and *Nocardia* spp. (*n* = 1) were also identified at a ≥ 99% identity match to the reference sequence. These genera, alongside *Precotella* spp. were also identified at varying percentage identities between 90 and 99%. Four of the eight samples identified with MTBC DNA also contained NTMs identified at the species level (heterogeneous mycobacterial population).

### Comparison of *Mycobacterium* spp. identified by sanger sequencing from culture and ONT tNGS of pooled PCR amplicons directly from oronasal swab DNA

Mycobacterial species identified from culture, followed by PCR speciation using Sanger sequencing, were compared to those obtained from culture-independent ONT tNGS. A total of 12/19 samples showed concordant results for the presence of mycobacterial DNA (Supplementary materials S3). Of these 12 samples, 2 were concordant for the presence of *M. avium* based on ≥99% identity match (S8B21N and S21B21M). In addition, ONT tNGS identified *M. novocastrense*, *M. neumannii*, and *M. elephantis* DNA alongside *M. avium* in one of these samples (S8B21N), while the other sample (S21B21M) also contained unspecified *Mycobacterium* spp. DNA that was not detected by Sanger sequencing. While Sanger sequencing of *hsp65* and *rpoB* amplicons was utilized to speciate mycobacteria in oronasal swab cultures, this method was unable to detect heterogeneous mycobacterial populations (Supplementary material S3). In contrast, the ONT tNGS of DNA extracted from oronasal swabs using the same targets, detected multiple samples with heterogeneous mycobacterial populations (Figure 2; Supplementary material S3). None of the 8 samples, which contained MTBC DNA based on ONT tNGS, had MTBC detected in swab cultures using Sanger sequencing. It is important to note that four culture sample DNA Sanger sequences were below the 90% coverage and identity match threshold for mycobacterial speciation, however, these were later found to contain mycobacteria with ONT tNGS.

## Discussion

This study aimed to culture-independently detect and characterize the mycobacterial species present in 19 African buffalo oronasal swabs collected from individuals with unexpected immune sensitization to mycobacterial antigens from historically *M. bovis-*free herds. These samples were selected based on identification of NTM DNA in oronasal swabs from an earlier study (19). In the current study, a panel of mycobacterial gene targets was used for PCR amplification, followed by long-read ONT amplicon sequencing to characterize the mycobacteriome. In order to identify all mycobacteria present in buffalo oronasal cavities, PCR targets were based on mycobacterial housekeeping genes (*rpoB, hsp65, gyrB*), which have been shown to be generally conserved across species but contain unique sequences that allow differentiation of *Mycobacterium* spp. (27). These results were compared to identified species generated from swab mycobacterial culture and speciation.

Conventional mycobacterial culture with speciation is the gold standard for detecting *M. bovis* infection in African buffalo and other species (8). However, this method has limitations, as shown by the lack of MTBC detection in buffalo oronasal swabs from a previous study (19). A potential explanation is the harsh decontamination processing of samples prior to culture, which could result in lower numbers of viable MTBC (12, 28, 29). Additionally, mycobacterial culture may change the microbial composition especially with long incubation times, favoring the more abundant, resilient, and often faster growing mycobacteria (15). This may lead to the underrepresentation or loss of mycobacteria that were initially present in lower numbers and slow growing, when characterizing microbial populations from culture (19, 28, 30). Therefore, in this study, culture-independent characterization of buffalo oronasal mycobacteriome was compared with mycobacterial species generated previously from paired buffalo oronasal swab cultures (19). The culture sequences were re-analyzed with stricter thresholds to ensure that the results obtained used the same criteria as the culture-independent sequences generated in this study. Sanger sequencing of *hsp65* and *rpoB* amplicons, using DNA extracted directly from the swabs, showed slightly higher numbers (*n* = 13) of samples identified with *Mycobacterium* spp. DNA compared to culture (*n* = 12), although no MTBC DNA was detected using either approach. The Sanger sequencing results identified the majority of *Mycobacterium* spp. as *M. avium*. This is not surprising since *M. avium* is ubiquitous in the environment, and likely contaminated the buffalo oronasal cavities.

An intriguing finding in this study was the identification of MTBC DNA in 8 of the 19 buffalo oronasal samples, 6 of which were speciated as *M. bovis*. None of the other previous analyses, using Sanger sequencing of PCR amplicons, indicated the presence of MTBC in these samples (19). Notably, in four of these samples, both MTBC and NTM DNA were present, which suggests that earlier results based on Sanger sequences missed paucibacillary MTBC. The low relative abundance of MTBC in these samples was not surprising, since these animals originated from historically *M. bovis* free herds. However, these results highlight the limitation of using Sanger sequencing for paucibacillary respiratory samples, due to its low coverage, and the inherent complexity of the sample type. In addition, Sanger amplicon sequencing may fail to identify co-occurring species, since strict criterion must be applied, which further impedes accurate speciation (31).

To overcome these disadvantages, ONT tNGS was employed to investigate the presence of mixed mycobacterial species, in DNA extracted directly from the oronasal swabs. One advantage of ONT tNGS is that it generates higher numbers and quality of reads, compared to Sanger sequencing (20). The increased depth of sequencing afforded by ONT tNGS facilitated a more thorough analysis of the mycobacterial species present in the samples (10, 11). This approach demonstrated that the majority of oronasal swabs contained multiple species of mycobacteria, including MTBC in eight cases, which is consistent with the complex nature of the oronasal mycobacteriome. Although MTBC DNA was identified using ONT tNGS, it is important to consider the historical *M. bovis* negative status of the herds, as well as the clinical assessment of the individuals when interpreting these findings. None of the animals showed clinical signs consistent with TB and initial testing was undertaken to meet regulatory requirements for transporting buffalo (48). The 19 buffalo swabs selected for further investigation were based on evidence of immunological responses to mycobacteria, which may be sensitization to MTBC or cross-reactivity to NTMs (19). Therefore, without evidence that the MTBC were viable, it is unclear whether the detection of DNA in eight oronasal swabs represented infection and shedding, residual MTBC DNA after clearing infection, or environmental contamination. Since cultures were negative for MTBC, the bacteria could have been non-replicating, or were present in too few numbers, which might have been eliminated during harsh decontamination steps prior to culture (12). When Cooke et al. (28) applied a similar approach to goat nasal swabs for MTBC detection, the culture-independent detection was higher than from cultures.

The source of MTBC in this study was unknown. Interestingly, the eight buffalo with MTBC DNA detected were from the same herd. Transmission of *M. bovis* in buffalo is believed to be primarily via infectious aerosols from shedding animals (6, 32). However, MTBC shed into the environment can remain viable for variable durations (33, 34). As animals graze, wallow, and create environmental aerosols with movement, exposure to environmental MTBC may occur (35, 36). There is growing evidence for indirect transmission of *M. bovis* between cattle and badgers sharing a contaminated environment as well as indirect interspecies spread in other ecosystems (36, 37). Therefore, one possible source of MTBC may have been previous environmental contamination associated with the historical presence of infected livestock, or current wildlife sharing the game reserve, although further investigation of environmental samples would be needed to determine epidemiological links to MTBC isolated from animals. In addition, culture would be required to confirm the presence of viable MTBC in buffalo and provide a source for whole genome sequencing to epidemiologically link cases.

This study had several limitations. The inability to acquire postmortem samples for gold standard confirmation of mycobacterial infection led to the alternative use of microbially complex antemortem samples (oronasal swabs). Previous reports suggest that MTBC shedding occurs sporadically from infected buffalo, and only some animals will be positive when using direct detection; thus, the use of respiratory samples could lead to false negative results (9). Buffalo in this study were suspected to be infected, based on immunological test results and presence of MTBC DNA in oronasal swabs. However, without evidence of mycobacterial viability, it was not possible to differentiate contamination or colonization, from infection. Importantly, it was not possible to rule-out false positive immunological results due to cross-reactivity to NTMs (19, 38). The paucibacillary nature of the samples may also indicate that sufficient antigen load may not have been able to cause immunosensitization, although low doses of *M. bovis* (1 cfu) can result in interferon gamma expression (39). Another limitation was the lack of PCR target amplification in a number of samples, especially for *hsp65*, *rpoB* and *gyrB*. The optimization of PCR for use with paucibacillary and complex samples remains a challenge. Alternative approaches, such as16S rRNA (16S) and shotgun metagenomic (SMg) sequencing, could have been employed. However, the amount of host DNA found in the directly extracted sample would have posed a challenge during SMg, sequestering the majority of much-needed reads (40). The speciation thresholds for 16S sequencing in mycobacteria have previously underperformed, given the genomic similarity found within the genus (41). Despite the higher resolution of ONT tNGS, a potential limitation is the reported error rates of this technology (42), although only high-quality sequences that could be assigned with high identity match with listed mycobacterial species were included in the analyses. The challenges of characterizing the diversity of microbial communities in complex samples suggests that new approaches are needed to interpret results when pathogenic mycobacteria are identified (17, 20).

In summary, the findings of this study demonstrate the utility of ONT tNGS to identify MTBC DNA in complex antemortem samples as an ancillary tool to conventional mycobacterial culture, especially in high value animals. The higher resolution and discriminatory power of ONT tNGS was used to investigate the apparent mycobacterial sensitization of individual African buffalo, based on diagnostic test results. Immunosensitization was hypothesized to have been due to low levels of MTBC, which conventional mycobacterial culture and subsequent PCR speciation did not detect. Culture-independent PCR and ONT tNGS, using DNA extracted from oronasal swabs, facilitated antemortem detection of MTBC in several African buffalo with evidence of immune sensitization. When used alongside mycobacterial culture, this technology may provide a comprehensive approach to characterizing the mycobacteriome in animals. Although methods may yield discordant results, their combined use allows for a more thorough understanding of the mycobacterial species present in a population, which may inform disease surveillance and control efforts.

## Data Availability

The datasets presented in this study can be found in online repositories. The names of the repository/repositories and accession number(s) can be found in the article/Supplementary material.
