# Supplementary material for: Culture-independent detection of Mycobacterium tuberculosis complex DNA using targeted next generation sequencing in African buffalo (Syncerus caffer) oronasal swabs in South Africa
Source: Front Vet Sci. 2025 Feb 7;12:1523628. doi: 10.3389/fvets.2025.1523628 (PMC11842352; doi:10.3389/fvets.2025.1523628)
Supplement: Supplementary file 3 [file Table_3.DOCX]

**Supplementary Material 1 (S1):** Supplementary Table 1 (S1): Sequencing statistics obtained from the ONT MinION Mk1C device using the R10.4.1 flow cell (FLO-MIN114) during the sequencing of the samples described in the manuscript.

**Supplementary Material 2 (S2):** Supplementary Material 2 (S2): Sequencing statistics obtained from the ONT MinION Mk1C device using the Flongle flow cell (FLO-FLG114) during the sequencing of the samples described in the manuscript.

**Supplementary Material 3 (S3):** Bacterial species identified using both culture and culture-independent methods described in the manuscript. Identification was subject to a coverage threshold of ≥ 90% with speciation thresholds assigned for genus and species level identification at 90-99% and ≥ 99%, respectively.
